# Patterns of anti-malarial drug treatment among pregnant women in Uganda

**DOI:** 10.1186/1475-2875-10-152

**Published:** 2011-06-06

**Authors:** Laura R Sangaré, Noel S Weiss, Paula E Brentlinger, Barbra A Richardson, Sarah G Staedke, Mpungu S Kiwuwa, Andy Stergachis

**Affiliations:** 1 Department of Global Health, University of Washington, Seattle, WA, USA; 2 Department of Epidemiology, University of Washington, Seattle, WA, USA; 3 Department of Biostatistics, University of Washington, Seattle, WA, USA; 4 Department of Clinical Research, London School of Hygiene and Tropical Medicine, London, UK; 5 Infectious Disease Research Collaboration, Kampala, Uganda; 6 Clinical Epidemiology, Makerere University, College of Health Sciences, School of Medicine Kampala, Uganda

## Abstract

**Background:**

Prompt use of an effective anti-malarial drug is essential for controlling malaria and its adverse effects in pregnancy. The World Health Organization recommends an artemisinin-based combination therapy as the first-line treatment of uncomplicated malaria in the second and third trimesters of pregnancy. The study objective was to determine the degree to which presumed episodes of uncomplicated symptomatic malaria in pregnancy were treated with a recommended anti-malarial regimen in a region of Uganda.

**Methods:**

Utilizing a population-based random sample, we interviewed women living in Jinja, Uganda who had been pregnant in the past year.

**Results:**

Self-reported malaria during the index pregnancy was reported among 67% (n = 334) of the 500 participants. Among the 637 self-reported episodes of malaria, an anti-malarial drug was used for treatment in 85% of the episodes. Use of a currently recommended treatment in the first trimester was uncommon (5.6%). A contraindicated anti-malarial drug (sulphadoxine-pyrimethamine and/or artemether-lumefantrine) was involved in 70% of first trimester episodes. Recommended anti-malarials were used according to the guidelines in only 30.1% of all second and third trimester episodes.

**Conclusions:**

Self-reported malaria was extremely common in this population and adherence to treatment guidelines for the management of malaria in pregnancy was poor. Use of artemether-lumefantrine combined with non-recommended anti-malarials was common practice. Overuse of anti-malarial drugs, especially ones that are no longer recommended, undermines malaria control efforts by fueling the spread of drug resistance and delaying appropriate treatment of non-malarial febrile illnesses. Improved diagnostic capacity is essential to ultimately improving the management of malaria-like symptoms during pregnancy and appropriate use of currently available anti-malarials.

## Background

The deleterious consequences of malaria infection during pregnancy for both the mother and her fetus have been widely reported [1-4]. The current World Health Organization (WHO) guidelines for the treatment of uncomplicated malaria in pregnancy recommend an artemisinin-based combination therapy (ACT) as the first-line treatment in the second and third trimesters of pregnancy [5]. Artemisinins are contraindicated during the first trimester of pregnancy due to embryo-foetal toxicity observed in studies of rats and rabbits [6-8] and limited safety data of early pregnancy use in humans [9,10]. However, the WHO does recommend first trimester use of ACT if the treatment is believed to be life-saving for the mother and other available anti-malarial drugs are considered unsuitable. Oral quinine plus clindamycin for seven days (or quinine monotherapy if clindamycin is not available) is the recommended first-line treatment in the first trimester of pregnancy for uncomplicated malaria [5]. Treatment with oral quinine is generally believed not to be toxic to the foetus, irrespective of trimester of use [5], although data are limited [5,9,11,12]. Clindamycin is often not affordable or available in most malaria endemic countries, and so quinine monotherapy is more common [12]. While chloroquine and sulphadoxine-pyrimethamine (SP) monotherapy are no longer recommended in sub-Saharan Africa for the treatment of uncomplicated falciparum malaria because of high levels of parasite resistance, their use remains commonplace [13,14]. Chloroquine is believed to be safe for use in all trimesters of pregnancy [9,10,15]. SP, a folic acid antagonist, is contraindicated during the first trimester of pregnancy because of the potential risk of cardiac anomalies and neural tube defects [10]; however, no association between SP exposure in early pregnancy and increased risk of congenital malformations has been reported [15].

In Uganda, malaria is thought to be the leading cause of morbidity and mortality, and accounts for almost half of all in-patient deaths among children less than five years of age [16]. The prevalence of placental malaria (based on PCR) was estimated at 26% for HIV uninfected women prescribed intermittent preventive treatment with SP (IPTp-SP) and 19% for HIV infected women prescribed daily trimethoprim-sulphamethoxazole [17]. The Ugandan Ministry of Health (MOH) recommends use of microscopy or rapid antigen testing for the diagnosis of malaria in pregnant women with symptoms of uncomplicated malaria [18]. However, diagnostic equipment and/or a trained technician are often unavailable in rural settings, resulting in a presumptive diagnosis of malaria based on clinical symptoms. Uncomplicated malaria generally presents with fever, malaise, and other non-specific symptoms; distinguishing malaria from other febrile illnesses based on clinical presentation is challenging [19-23]. Presumptive treatment of malaria based on fever occurs commonly, leading to overuse of anti-malarial drugs and delays in seeking treatment for other febrile illnesses [20,24-27].

In Uganda, artemether-lumefantrine (AL), an ACT, is the first-line recommended treatment for uncomplicated malaria. While the Ugandan MOH changed their policy regarding the treatment of uncomplicated malaria in pregnancy from chloroquine plus SP to ACT in 2004 based on the WHO recommendations[18], this policy was not implemented until 2006. During the first half of 2006, all government, private, and not-for-profit health care facilities received AL free-of-charge [28]. These supplies were followed by in-service trainings and the provision of wall-charts [28]. AL was also meant to replace chloroquine plus SP in the national home-based management of fever program. However, since 2006, national shortages of AL have challenged the implementation of this policy and proper treatment of malaria in Uganda [28-30].

The purpose of the present analysis was to determine the degree to which presumed episodes of uncomplicated symptomatic malaria in pregnancy were treated with a recommended anti-malarial regimen in a region of Uganda.

## Methods

### Selection of study participants

Between November, 2008 and January, 2009, home-based interviews were conducted among a simple random sample of 500 female residents of Kibibi and Namizi parishes in Budondo-sub county of Jinja District, Uganda, to determine patterns of self-reported malaria episodes and self-reported anti-malarial drug use during pregnancy. The parent study ascertained use of insecticide treated nets (ITNs) and intermittent presumptive therapy (IPTp) with SP during pregnancy, as well as possible factors associated with use. Women who had a pregnancy within the past 12 months that lasted until at least the third trimester, regardless of pregnancy outcome, between the ages of 15 and 49 years, were eligible to participate. Details of the study population and procedures have been described elsewhere [31].

### Study site

Sampling was based on census data completed in November 2008 by the Ugandan Malaria Surveillance Project. Namizi and Kibibi parishes are comprised of 21,681 residents living among 16 rural and peri-urban villages, of whom 4,654 were females aged 15-49 years, and 867 of these women reported having been pregnant in the previous 12 months. One public health center that provides antenatal care is located in each parish; Kibibi has a level II facility and Namizi has a level IV facility. The primary economic activity in this area is farming, with sugar cane and coffee being the main cash crops.

The average annual entomological inoculation rate in Jinja is 6 infective bites per person per year [32]. Malaria transmission in this area is considered meso-endemic, defined by transmission occurring throughout the year with peaks during two rainy seasons. A cross-sectional parasitological survey identified a prevalence of the *Plasmodium falciparum *parasite of 15% among children 1-9 years of age in 1999 [33].

### Administration of the survey

A pregnancy history calendar was generated for each woman to record episodes of self-reported malaria, any anti-malarial use during pregnancy, and antenatal care (ANC) visits. Additionally, when asking specifically about SP and AL, women were shown photographs of SP and AL packaging and the corresponding tablets for the most common formulations of these drugs available in the area.

In this setting, non-artemisinin anti-malarials were available from privately owned drug shops and other non-clinical sources, making presumptive self-treatment unaccompanied by medical consultation possible. Because one of the aims of the project was to identify factors associated with use of IPTp with SP, for each ANC visit the woman attended, she was asked if SP was offered and administered by the health facility. The source of non-SP anti-malarial medications (e.g. health center, drug shop, community health worker, friend) was not ascertained. Gestational age by trimester was estimated by subtracting an average pregnancy length of 40 weeks from the child's date of birth unless other information was provided to suggest the pregnancy was of a shorter or longer duration. This information allowed for the identification of the calendar month associated with each trimester of pregnancy.

### Data management

A pre-tested interviewer-administered structured questionnaire was used to collect participant responses. The questionnaire ascertained information on socio-demographic characteristics, socio-cultural factors, obstetric history, knowledge and attitude about malaria, antenatal clinic use, use of ITNs and IPTp, self-reported episodes of malaria and anti-malarial drug use. Wherever possible, questions were taken from those used in large international surveys, including the Uganda Demographic Health Survey, and the Malaria Indicator Survey [34,35]. The questionnaire was translated from English to Lusoga.

### Definitions

Self-reported malaria was ascertained by asking the woman if she believed she was ever sick with malaria during the index pregnancy. If a study subject reported yes, then the timing and treatment of each presumed episode was recorded. Because the policy of presumptive treatment of malaria based on clinical symptoms is common in this area, and the public health centers in these parishes lack diagnostic equipment, it was determined that soliciting additional information regarding a health provider's diagnosis or laboratory test would not greatly improve the validity of this assessment. All possible treatments utilized were recorded for each episode and were captured as: AL, SP, quinine, chloroquine, paracetamol, herbs, no treatment, or other if none of the above. Treatments were compared to anti-malarials currently recommended by the Ugandan MOH, stratified by first and second/third trimesters of pregnancy. Current recommendations were enacted as policy in 2005, and pregnancies included in the study occurred between 2007 and 2009.

### Statistical analysis

Analyses were performed using Stata version 11.0 (College Station, Texas, USA). Patterns of use of anti-malarials drugs in response to self-reported episodes of malaria were determined and reported as frequencies. Primary analyses calculated the proportion of self-reported malaria episodes treated with a WHO recommended drug regimen, stratified by trimester of pregnancy.

### Ethical approval

The study was approved by the Makerere University Research and Ethics Committee, the Uganda National Council for Science and Technology, and the University of Washington Human Subjects Division. All participants provided written informed consent.

## Results

### Characteristics of the study population

Six hundred twenty nine households were visited between November 2008 and January 2009 to identify 500 eligible women. All of the potential study subjects agreed to participate. Eligibility and participation of study subjects have been published elsewhere [31]. The demographic characteristics of the study population are shown in Table 1. Stillbirth occurred in seven of the index pregnancies, and the remaining pregnancies ended in a live birth. Nearly all women (99%) attended ANC at least 1 time, 95% with 2 or more visits, 73% had 3 or more visits, and 34% with the recommended four or more visits during pregnancy. The mean number of ANC visits was 3.2, and 86% of women initiated ANC during the 1^st ^or 2^nd ^trimester.

**Table 1:** Characteristics of the study population

Characteristic	Total Population n = 500
Age (years); n (%)	
≤18 years	50 (10.0)
19 - 24 years	204 (40.8)
25 - 34 years	182 (36.4)
≥35 years	64 (12.8)
Married; n (%)	451 (90.2)
Education; n (%)	
None	34 (6.8)
Primary	350 (70.0)
Secondary/Postsecondary	116 (23.2)
Religion; n (%)	
Christian-based	305 (61.0)
Muslim	195 (39.0)
Village type; n (%)	
Rural	372 (74.4)
Peri-Urban	128 (25.6)
Number of births; n (%)	
1	111 (22.2)
2-3	152 (30.4)
4-5	111 (22.2)
≥6	126 (25.2)
History of miscarriage; n (%)	85 (17.0)
History of stillbirth; n (%)	23 (4.6)
Birth location; n (%)	
Health facility	367 (73.4)
Home	81 (16.2)
Other	52 (10.4)
Knowledge of malaria score; n (%)	
High	292 (58.4)
Low	208 (41.6)
# ANC visits; n (%)	
0	6 (1.2)
1	23 (4.6)
2	106 (21.2)
3	195 (39.0)
≥4	170 (34.0)
Month of ANC initiation; mean (sd)	4.8 (1.6)
Walking time to nearest ANC, minutes; mean (sd)	69 (44.2)
Waiting time at ANC, minutes; mean (sd)	58 (47.7)

The self-perceived risk of malaria during pregnancy was high, with 70% of women responding they were specifically worried about getting sick with malaria during their most recent pregnancy. Miscarriage/stillbirth was the most feared consequence of malaria in pregnancy reported by women (50%), followed by maternal death (16%), and the baby being adversely affected (12%).

### Occurrence of self-reported malaria

Self-reported malaria during the index pregnancy was reported by 67% (n = 334) of participants (Table 2). Multiple self-reported malaria episodes were also common, with 37% of women reporting two or more malaria episodes. A total of 637 episodes were reported in the cohort. Among women with self-reported malaria, the first reported malaria episode occurred most frequently during the 2^nd ^trimester (46%), followed by the 1^st ^trimester (36%).

**Table 2:** Self-reported malaria during pregnancy

Characteristic	TOTAL (n = 500 women)
Women with self-reported malaria during index pregnancy; n (%)	334 (66.8)
Number of self-reported malaria episodes per woman; mean (sd)	1.3 (1.2)
0 episode; n (%)	166 (33.2)
1 episode; n (%)	149 (29.8)
2 episodes; n (%)	95 (19.0)
3 episodes; n (%)	67 (13.4)
4 episodes; n (%)	18 (3.6)
5 episodes; n (%)	5 (1.0)
	
Total number of self-reported malaria episodes during index pregnancy; n	637

### Self-reported use of anti-malarials during pregnancy

Among women with self-reported malaria, 94% reported using an anti-malarial drug at least once to treat malaria during the index pregnancy. This represents 63% of all women interviewed.

Among the 637 self-reported episodes of malaria during the index pregnancy, an anti-malarial drug was used for treatment in 85% of the episodes. Use of anti-malarials was stratified by trimester of pregnancy and categorized by currently recommended treatments (Table 3). Use of a currently recommended treatment in the first trimester was uncommon: only 5.6% of first trimester episodes were treated with quinine. A contraindicated anti-malarial drug (SP and/or AL) was involved in 70% of first trimester episodes. Use of AL alone or in conjunction with another anti-malarial was the most common regimen in the first trimester (42%), followed by SP monotherapy (23%). A stratified analysis showed no difference between the proportion of women using a recommended treatment in the first trimester between primigravid and multigravid women (p = 0.4).

**Table 3:** Proportion of self-reported treated malaria episodes in which an anti-malarial currently recommended by the Ugandan MOH was used, by trimester of pregnancy

1^st ^Trimester	TOTAL (n = 126 episodes*)	(95% CI)^+^
Currently recommended		

Quinine monotherapy; n (%)	7 (5.6)	(2.5, 10.7)

Not currently recommended	119 (94.4)	(89.3, 97.5)

SP monotherapy; n (%)	29 (23.0)	
Chloroquine monotherapy; n (%)	18 (14.3)	
SP+Chloroquine; n (%)	6 (4.8)	
Any AL; n (%)	53 (42.1)	
AL alone; n	26	
AL +1 other anti-malarial^1^; n	21	
AL + 2 or more other anti-malarials^2^; n	6	
Other; n (%)	13 (10.3)	

**2^nd ^or 3^rd ^Trimester**	TOTAL (n = 478 episodes*)	

Currently recommended	144 (30.1)	(26.1, 34.3)

AL alone; n (%)	113 (23.6)	
Quinine monotherapy; n (%)	28 (5.9)	
AL + quinine**	3 (0.6)	

Not currently recommended	334 (69.9)	(65.6, 73.9)

SP monotherapy; n (%)	132 (27.6)	
AL +1 other anti-malarial^3^; n	78 (16.3)	
Chloroquine monotherapy; n (%)	31 (6.5)	
SP+Chloroquine; n (%)	23 (4.8)	
AL + 2 or more other anti-malarials^4^; n	13 (2.7)	
SP+Quinine	5 (1.1)	
Other; n (%)	52 (10.9)	

Any use of a currently recommended anti-malarial***; n (%)	240 (50.2)	(45.7, 54.7)

Any use of AL; n (%)	207 (43.3)	
Any use of quinine; n (%)	33 (6.9)	

AL: Artemether-lumefantrine

SP: Sulphadoxine-pyrimethamine

^+^95% Confidence Interval around the proportion

*Excludes 33 episodes that were not treated; 1^st ^trimester = 8 episodes; 2^nd ^trimester = 25 episodes

**Possibly correct depending on clinical circumstances

***Includes use of recommended drug combined with a non-recommended drug

^1^AL plus: SP (n = 16); Chloroquine (n = 3); Quinine (n = 2)

^2^AL plus: SP & Chloroquine (n = 4); SP & Quinine (n = 1); SP & Chloroquine & Quinine (n = 1)

^3^AL plus: SP (n = 71); Chloroquine (n = 7)

^4^AL plus: SP & Chloroquine (n = 11); SP & Quinine (n = 2)

Half of all second and third trimester episodes (240/478) were treated with a currently recommended anti-malarial (AL or quinine). However 40% of episodes treated with a recommended anti-malarial (96/240) were also treated with an additional, non-recommended anti-malarial, either before, after, or concurrently with the recommended anti-malarials (Table 3). Recommended anti-malarials were used according to the guidelines in only 30.1% of all second and third trimester episodes. Precise data on the sequence of anti-malarials during treatment of individual episodes of self-reported illness are not available. SP, which was not recommended for treatment of symptomatic malaria owing to widespread drug resistance, was used alone or in combination in 33% of the episodes. There was no difference between the proportion of women using a recommended treatment in the second/third trimester of pregnancy between primigravid and multigravid women (p = 0.6).

## Discussion

These results suggest that self-reported malaria was extremely common in this population; nearly two-thirds of women experienced at least one episode of illness during their most recent pregnancy. Adherence to treatment guidelines for management of malaria in pregnancy was poor. Almost all self-reported malaria episodes occurring during the first trimester of pregnancy were treated with a non-recommended regimen and 70% included a drug that is contraindicated in pregnancy. In second and third trimesters, only one-third of episodes were treated with an anti-malarial regimen according to the guidelines. Use of AL combined with non-recommended anti-malarials was common practice.

To minimize adverse pregnancy outcomes related to malaria, timely and effective treatment is essential. However, only a limited number of anti-malarial drugs available are deemed safe for use in pregnancy. The lack of safe and effective drugs for treatment of malaria in pregnancy is a considerable problem that is exacerbated for malaria episodes occurring in the first trimester as even fewer drugs are recommended during this embryo-sensitive period. Use of oral quinine is particularly problematic considering its marked side-effects, poor adherence, and a long complicated treatment course requiring a dose be taken every 8 hours for 7 days [10,12,36,37]. Furthermore, *P. falciparum *resistance to quinine has been reported in Southeast Asia, which may eventually limit the effectiveness of this regimen [38]. Therefore, first trimester alternatives are needed. Currently, ACT is contraindicated in the first trimester due to safety concerns for potential teratogenicity based on the severe embryo-fetal toxicity observed in rats and rabbits [6-9]. Recent modeling suggests the probability is 12% that an embryo will encounter an inadvertent exposure to artemisinins during the embryo-sensitive period (between week four and ten of gestation) in areas where adults receive on average one three-day treatment with an ACT per year [39]. Monitoring inadvertent and intended first trimester exposures to ACT using pharmacovigilance systems such as pregnancy exposure registries, may eventually provide enough safety evidence in humans to warrant their use in the first trimester [40]. The absence of adequate safety data, especially in the first trimester, is an important obstacle to malaria control in pregnancy [41].

Efficacy data from a recent randomized trial conducted on the Thai-Burmese border reported that 63.4% (95% CI: 46.9%, 77.4%) of pregnant women with symptomatic uncomplicated *P. falciparum *malaria during the second or third trimesters who were treated with a supervised 7-day course of quinine monotherapy were cured, based on polymerase chain reaction corrected parasite clearance at day 63 of follow-up or delivery [42]; this was considerably lower than the proportion of cures in women treated with three days of artesunate-atovaquone-proguanil (94.9%; 95% CI: 81.37%, 99.11%). An earlier randomized trial, comparing artesunate versus quinine plus clindamycin for the treatment of uncomplicated *P. falciparum *malaria by the same team in the same area, reported no difference in efficacy with 100% of the women in each arm cured [43]. Safe and effective anti-malarial use after the first trimester of pregnancy is limited to ACT in most geographic areas, although the available safety data are limited to 1,500 pregnancies exposed to artemisinin compounds in the 2^nd ^and 3^rd ^trimesters of pregnancy; in these pregnancies there were no adverse events reported for the mother or the fetus [5,9,44-46].

When the symptomatic patient is indeed parasitaemic, continued use of ineffective drugs may select for drug resistant strains [47]. The extent of the problem of continued use of ineffective anti-malarials was demonstrated in our population, in which 44% of all self-reported malaria episodes were treated using SP and/or chloroquine. Anti-malarial drug-efficacy studies conducted in Jinja between December 2002 and May 2004 reported a risk of treatment failure of 50% for SP plus chloroquine among children aged six months to five years with uncomplicated malaria [48]. Women receiving SP for treatment of malaria in Jinja are at a high risk of treatment failure, and subsequent morbidities associated with severe disease.

The most recent guidelines for the treatment of malaria from WHO recommend that, in all settings, clinical suspicion of malaria should be confirmed with a parasitological diagnosis, except in places where parasitological diagnosis is not possible [5]. However, in most parts of Africa where malaria is endemic, parasitological diagnosis is indeed not possible, and so presumptive treatment continues. Considering that malaria typically presents with non-specific symptoms of fever and malaise that are indistinguishable from several other febrile illnesses common in the same areas, this practice of empirical treatment results in substantial overuse of anti-malarial drugs [20,24-27] and often delays the diagnosis of other febrile illnesses leading to poor clinical outcomes and heightened mortality [13,47]. A recent study from Mozambique of pregnant women seeking care for malaria-like symptoms found only 27% of women with fever were parasitaemic [24]. These data are similar to other studies conducted in adult and child populations in Africa reporting only 24-32% of patients with malaria-like symptoms had malaria, defined by fever with parasitaemia [25,26,49]. The lack of safety data on most anti-malarials recommended for use in pregnancy, particularly for first trimester drug exposures when organogenesis occurs [5,9,10,50], highlights the importance of limiting the use of anti-malarial drugs during pregnancy to women whose symptoms are caused by malaria. With the advent of rapid diagnostic testing (RDTs), there is hope that access to parasitologically-based diagnoses will improve, leading to an eventual decrease in overuse of anti-malarials and drug resistance. However, even in clinics where RDTs are available, health workers persist in giving anti-malarials despite negative results [27,51], highlighting the strength of the widespread belief that fever equals malaria and lack of clinical capacity to identify alternative diagnoses. Dedicated resources and political commitment will be needed to expand use and acceptance of RDTs, but ultimately this technology has the potential to result in a cost-savings by avoiding use of expensive ACT-based treatments on people who do not have malaria [52]. It was recently estimated that for every billion dollars in subsidy for anti-malarial drugs, around $500 to $900 million dollars are spent on treatments for people who do not have malaria [52].

This study has several limitations. Data on parasitologically confirmed cases of malaria were unavailable and, therefore, we were unable to distinguish which women had malaria and which ones did not. Data on specific symptoms were also unavailable and therefore uncomplicated cases could not be distinguished from severe cases, and so we were unable to determine which cases could be exempted from the recommendations that are based on uncomplicated malaria. The location where anti-malarial drugs other than SP came from was not ascertained and, therefore, the extent to which self-treatment occurred in this population is not known. The dispensing of AL was expected to be limited to health facilities due to the higher cost associated with this medication and the general lack of availability of AL in the private sector. Among women who used more than one anti-malarial per episode of malaria, it is unknown if the same provider prescribed the drugs for each episode, or if this represents women seeking care from multiple providers, or if self-treating with serial drugs from multiple shops was occurring. Furthermore, the availability of an appropriate first-line anti-malarial treatment in the health facility at the time the woman received an alternate treatment is unknown. Therefore, caution is needed when interpreting these results in relation to health providers. Women with more severe malaria-like symptoms may have been more likely to recall the details of this event. However, considering the majority of women believed they had malaria at least once during the index pregnancy, recall bias is unlikely to be significant. Women did not appear to have problems to recall use or timing of anti-malarial drugs during pregnancy. This may be expected considering there are only a limited number of drugs available for the treatment of malaria in pregnancy, and that women are quite experienced with the treatment of malaria based on the high burden of disease in this community. The process of gestational age estimation may have resulted in non-differential misclassification of trimester of anti-malarial use making frequencies equally as likely to be under-estimated in each trimester.

To improve recall, interviews were assisted with a pregnancy history calendar in which each pregnancy was mapped out over time on the calendar to record episodes of self-reported malaria, any use of SP or other anti-malarials during pregnancy, and ANC visits. We sought to improve the reliability of data related to anti-malarial drug use in response to self-reported episodes of malaria by asking multiple questions related to each throughout the interview. Additionally, when asking about SP and AL, we showed women photographs of SP and AL packaging and the corresponding tablets for the most common formulations available in the area. As previously reported, the reliability of self-report of SP use was verified using ANC cards [31]. The concordance between self-reported SP doses and ANC card was high (Pearson's rho = 0.93). In an attempt to decrease misclassification between SP used for IPTp and SP used as treatment, women were asked if they believed they were sick with malaria at the time they took each dose of SP.

## Conclusions

Overuse of anti-malarial drugs, especially ones that are no longer recommended, undermines malaria control efforts by fueling the spread of drug resistance and delaying appropriate treatment of non-malarial febrile illnesses. Unnecessary exposures to anti-malarials in pregnancy are particularly of concern given the lack of human safety data on these drugs. In addition to intensified measures to reduce malaria transmission, improved diagnostic capacity is essential to ultimately improving the management of malaria-like symptoms during pregnancy and appropriate use of currently available anti-malarials. However, improving diagnostic capacity will require additional resources and changes to the existing health care infrastructure.

## Competing interests

The authors declare that they have no competing interests.

## Authors' contributions

LS conceived of the study, and led the study design, protocol development, data collection, analysis and writing of the manuscript. NW and AS participated in the study design. SS and MK assisted with the coordination of the data collection. NW, AS, PB, BR, SS, and MK contributed to the analysis plan, participated in the interpretation of results and preparation of the manuscript. All authors read and approved the final manuscript.
